# Protocol for efficient recovery of high-quality DNA from microbiome of marine invertebrates

**DOI:** 10.71150/jm.2507003

**Published:** 2025-09-30

**Authors:** Yeong-Jun Park, Jae Kyu Lim, Yeon-Ju Lee, Kae Kyoung Kwon

**Affiliations:** 1 Marine Bioresources & Biotechnology Division, Korea Institute of Ocean Science and Technology, Busan 49111, Republic of Korea; 2 KIOST School, University of Science and Technology, Daejeon 34113, Republic of Korea; 3 Jeju Bio Research Center, Korea Institute of Ocean Science and Technology, Jeju 63349, Republic of Korea

**Keywords:** microbiome, DNA extraction, marine invertebrates, purification

## Abstract

Marine organisms often form symbiotic relationships with various microorganisms to adapt and thrive in harsh environments. These symbiotic microbes contribute to host survival by providing nutrition, modulating the hosts’ immune system, and supporting overall physiological stability. Advances in high-throughput sequencing technologies have enabled a deeper understanding of the structure and function of symbiotic microbial communities, as well as host-microbe interactions. Notably, symbiotic bacteria associated with marine invertebrates such as corals and sponges are recognized as a potential source of useful bioactive compounds, including antibiotics and enzymes. However, obtaining high-quality microbial DNA from host tissues still remains a technical challenge due to the presence of unknown substances. This study focuses on optimizing sample preparation and DNA extraction procedures and additional purification to improve the recovery of microbial DNA while minimizing host DNA contamination. Comparison between several methods was conducted using sponge samples to evaluate DNA quality and microbial recovery. A sample designated as 2110BU-001 was collected from the east coast of the Republic of Korea and used for culture-independent microbial cell isolation. Total bacterial DNA was extracted by using a manual Phenol-Chloroform protocol and three commercial kits. DNA extracted using the standard manual method showed both the highest yield and the largest fragment size. However, PCR (Polymerase chain reaction) test showed that quality of manually extracted DNA was not enough for sequencing. Therefore, the quality of DNA was improved through additional purification steps. Briefly, host eukaryotic cells were removed by mechanical process and almost only bacterial DNA was successfully obtained by combination of manual extraction method and further purification processes. The established protocol was successfully introduced to extraction of metagenomic DNA from mussel and jellyfish microbiomes, indicating that it can be widely applied to various marine organisms.

## Overview

The ocean is the largest and most diverse ecosystem on earth, containing a great biological and chemical diversity of organisms like eukaryotic marine organisms, viruses, prokaryotes, and fungi (Das et al., 2006). These organisms are deeply involved in marine primary production, food web, carbon pump and biogeochemical cycles (Trombetta et al., 2020). In addition, microbiomes play an essential roles in maintaining health, development, and environmental interactions of their hosts (Ma et al., 2023). Advances in high-throughput sequencing have enabled omics studies to reveal host-symbiont relationships, from the human gut to marine invertebrates. Microbiome research provides insight into the microbial diversity and functions on hosts or environments. In addition to their ecological importance, microbiomes are a promising source of novel bioactive compounds with industrial potential. For example, sponges (phylum *Porifera*) are well known to produce diverse secondary metabolites such as anticancer and anti-infective therapeutic agents (Sabdono and Radjasa, 2008). Furthermore, marine sponge-derived biologically active compounds have been identified to act as antibacterial, antiviral, anti-malarial, and anti-fungal agents (Varijakzhan et al., 2021). These compounds are structurally similar to metabolites of their associated microorganisms including bacteria (Proksch et al., 2002; Radjasa et al., 2007a, 2007b), and sponge-derived compounds known to date, including polyketide types, are likely to be produced by symbionts (Kurnia et al., 2017). Therefore, it is important to understand the role of marine sponge-associated microorganisms. For massive production of useful compounds, a number of studies about synthesis and other fields of application are progressing. Especially, molecular biological approaches have provided unique insight of uncultured microbial communities from soils, waters, and various organisms (Miller et al., 1999). For example, metagenomics is a powerful method to study entire gene pool of communities (Gaur et al., 2019), and information of interaction between host and symbionts can be obtained via the metagenomic approaches. In addition, genetic information for biosynthesis of bioactive compounds can be obtained. Especially, marine sponge-associated microorganisms such as *Actinomyces*, *Streptomyces* and fungi produce many secondary metabolites with diverse chemical structures and biological properties for drug developments and they are the potential resources for novel enzymes (Dashti et al., 2014; Su et al., 2015; Wei et al., 2011). Despite this potential, microbiome research still faces significant methodological challenges including difficulty in selectively extracting microbial DNA without host contamination (Santos et al., 2013), incomplete cell lysis, coextraction of enzymatic inhibitors, and loss, degradation, and damage of DNA (Miller et al., 1999). Therefore, many studies have been conducted and improved on DNA extraction techniques to solve these difficulties. When extracting DNA from environmental samples such as soil, water, and organisms, cell extraction (recover of cells from environmental samples) methods have also been regarded as an important process. In this study, we separated the bacterial cells from marine sponges excluding host eukaryotic cells by mechanical process, extracted total bacterial DNA, and improved purity by removing contaminants via modified DNA purification methods. Additionally, it was confirmed that it could be applied not only to sponges, but to invertebrates such as mussels with complex organs. This study presents a sample preparation method for various omics analyses.

## Applications

Metagenomic approaches have become essential tools for studying the taxonomic composition, functional potential, and host-microbe interactions within complex microbial communities, especially those associated with marine organisms (Ejaz et al., 2024). By providing comprehensive genetic information including from uncultivable microorganisms, metagenomic analysis enables the identification of symbiont-derived genes potentially involved in the biosynthesis of bioactive secondary metabolites (Wu et al., 2022). These capabilities offer valuable insights into the ecological roles of symbiotic microbes and their contribution to host physiology. For example, metagenomic analysis of sponge-associated microbiomes may reveal functional genes for biosynthesis of novel bioactive compounds (Nakashima et al., 2016). These compounds exhibit a broad range of biological properties, including antibiotics, immunosuppressants, antidiabetics, hormone (ion channel or receptor), antagonists, anti-cancer drugs, and even as agricultural agent (Sun et al., 2019). Moreover, microbiome research is not limited to the discovery of bioactive compounds, but extends to understanding how microbial communities influence the host’s life cycles. For instance, jellyfish-associated microbiomes may have functional genes that contribute to the unique physiological properties of the host, such as metamorphosis (Peng et al., 2024). As another example, metagenome analysis of gill symbionts of large deep-sea mussels can identify environmentally specific adaptive genes, enabling studies on how host symbionts can adapt to environmental stress and influence their hosts (Sun et al., 2025).

**Fig. 1.**
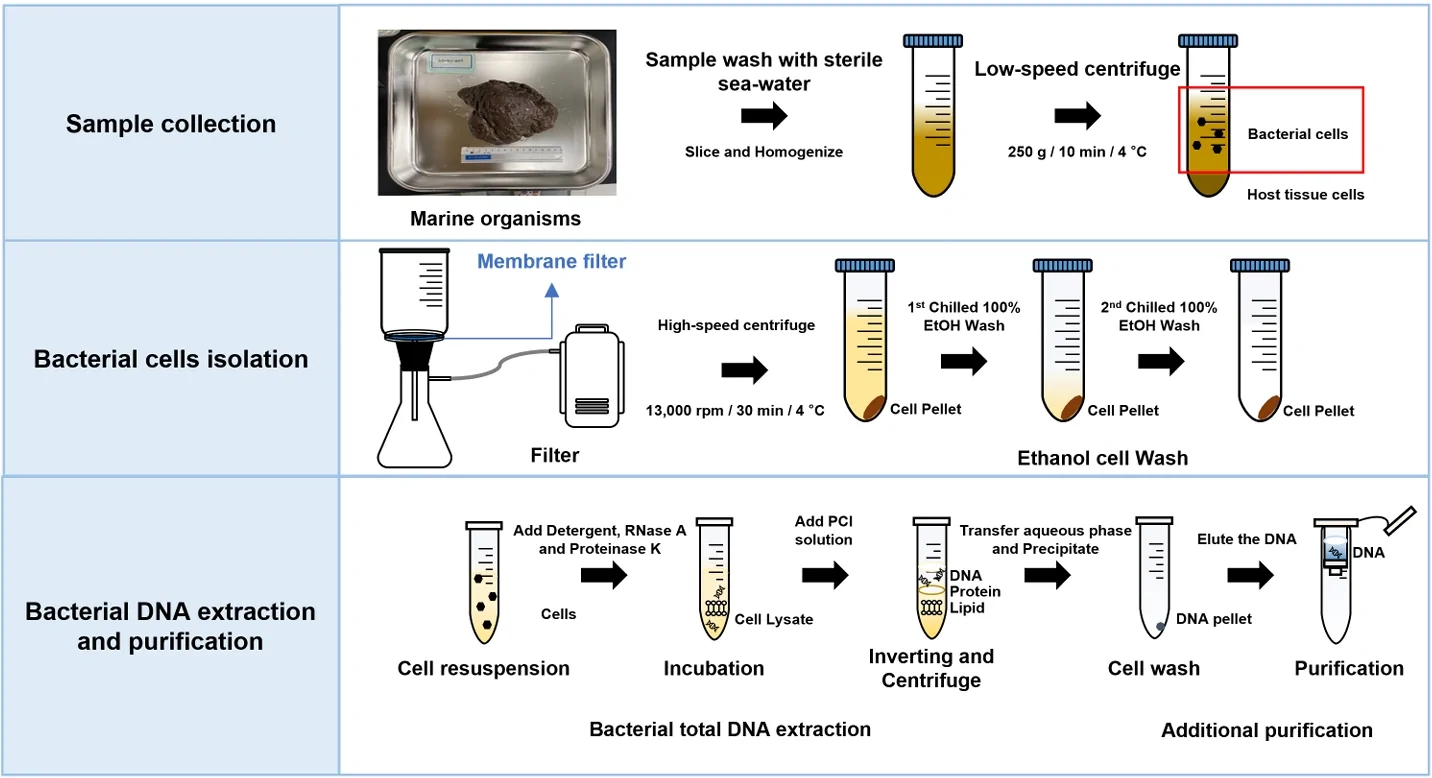
Overview of metagenomic DNA extraction from marine organisms. This figure shows sample preparation, host tissue debris removal, and metagenomic DNA extraction and purification.

However, the success of metagenomic studies largely depends on the quality of extracted DNA. Host DNA contamination and biased microbial recovery can reduce the accuracy of subsequent analyses. The protocol described in this study was designed to address these limitations by enabling selective isolation of symbiotic microbial cells and the extraction of high-purity metagenomic DNA while minimizing host DNA contamination. The procedure is summarized in Fig. 1. Through a combination of differential centrifugation, membrane filtration, and additional DNA purification steps, this protocol effectively removes host-derived materials and PCR inhibitors, as demonstrated by PCR-based validation. When DNA yields and purity were compared with three commercial bacterial gDNA extraction kits, the proposed method was confirmed to exhibit excellent performance in recovering high-quality microbial DNA.

While the method was developed using sponge-associated microbiome, it has also been successfully applied to mussels and jellyfish belonging to other phyla. This broad applicability makes the protocol a valuable tool for studying the microbiomes of diverse marine invertebrates and has potential for use in ecology, biotechnology, and natural product discovery research. The protocol presented in this study can be applied to sample preparation for bacterial analysis, as in the examples above, and conversely, it can also be applied to the preparation for DNA and RNA analysis of hosts by excluding bacteria.

## Methods

### Sample preparation and bacterial cell separation

A marine sponge, designated 2110BU-001, was collected by SCUBA diving in the East Sea (longitude 35°13’39’’N, latitude 126°33’48’’E) in 2021. The sponge was immediately transported to the laboratory on ice. The sample surface was washed twice with sterile seawater and then stored in 100% ethanol at –80°C until bacterial cell collection and DNA extraction. Frozen sponge sample was dissociated in sterile sea water at 4°C for 15–18 h. A 90 g sample was placed in a 250 ml beaker containing 150 ml sterile seawater and sliced with sterile mayo scissors or scalpel. The sliced sample was homogenized, resuspend in sterile seawater, and filtered with a 100 μm pore size sieve (Cisa, Spain). The filtered sample was centrifuged at 250 g for 10 min at 4°C and the supernatant was carefully transferred into a new tube to remove host tissue debris and any other contaminants. The obtained supernatant containing bacteria was filtered twice through 3 μm pore size membrane filter (MF-Millipore Membrane Filters, Merck). Obtained samples were washed using 100% chilled ethanol until the color was removed by repetition of suspended and centrifugation and stored at –80°C until DNA extraction. Mussel sample, designated 21MS-02, was collected from a mussel farm in Masan (longitude 35°12’08’’N, latitude 128°58’63’’E) in 2021. Jellyfish samples were collected from Sea Life Busan aquarium (Korea). Symbiotic microorganisms were separated using the same method as for sponge sample above, and bacterial DNA was extracted.

#### 1. Total DNA extraction and evaluation of DNA

**Fig. 2.**
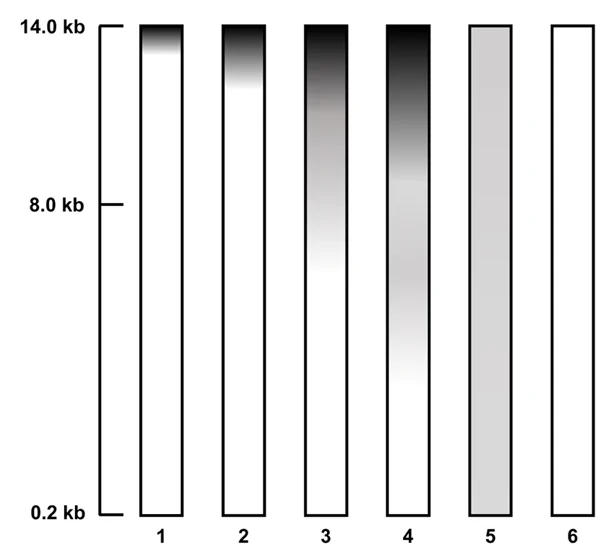
Degradation scores for DNA quality, modified from Amos and Hoelzel (1991).

Bacterial DNA isolated from a marine sponge (2110BU-001) was analyzed by agarose gel electrophoresis at 100 V for 50 min in 0.6% agarose gel with 0.5X TAE buffer. The quantity and purity of DNA were determined by measuring the OD_260/280_ value using a NanoDrop Spectrophotometer ND-2000 (Thermo Fisher Scientific, USA). The quality of genomic DNA was evaluated using the Amos and Hoelzel score (Amos and Hoelzel, 1991) (Fig. 2). For example, scores from 1 to 6 were given depending on the degree of DNA degradation. PCR test was performed on DNA samples obtained by each extraction method to confirm whether the purity was enough for sequencing. Each PCR reaction contained universal bacterial primer set 27F (5’-AGA GTT TGA TCM TGG CTC AG-3’) and 1492R (5’-GGT TAC CTT GTT ACG ACT T-3’) for 16S rRNA gene (Frank et al., 2008) and EukA (5’-AAC CTG GTT GAT CCT GCC AGT-3’) and EukB (5’-TGA TCC TTC TGC AGG TTC ACC TAC-3’) for eukaryotic 18S rRNA gene fragments (Medlin et al., 1988). The 30 μl PCR mixtures contained 50 ng of DNA as a template, a 0.2 µM concentration of deoxynucleotide triphosphate (dNTPs), 1X Ex-Taq buffer (TaKaRa), 0.2 µM (each) primer, and 1.25 U of Ex-Taq DNA polymerase (TaKaRa). All PCR reactions were run by thermocycler (Biometra TAdvanced 96SG, analyticjena) as follows: 94°C for 5 min, followed by 30 cycles of 94°C (20 s), 52°C (30 s), and 72°C (2 min), a final elongation step of 72°C for 5 min. PCR products (approximately 1.4 kb for 16S rRNA gene and 1.8 kb for 18S rRNA gene) were electrophoresed on a 0.8% agarose gel with GeneRuler 1 kb DNA Ladder (Thermo Fisher) as a size marker, and run in Tris-acetic acid-EDTA (TAE) buffer at 120 V for 25 min. Ethidium bromide (EtBr) solution (0.5 µg/µl) was used for staining.

##### 1.1. Standard manual extraction

Bacterial cells separated from marine sponge sample (2110BU-001) were centrifuged at 13,000 rpm for 10 min at 4°C. The pellet was washed twice with 100% chilled ethanol and TNE buffer (100 mM Tris-HCl, 100 mM NaCl, 0.5 mM EDTA; pH 8.0). The washed pellet was resuspended in 500 µl of TNE buffer. For cell lysis, 10% sodium lauryl sarcosine (Final 1%), 10% SDS solution (Final 1%), and 10 mg/ml RNase A (Final 0.1 mg/ml) were added, mix gently by inverting 10 times and incubated at 37°C for 15 min in water-bath. Add proteinase K (20 mg/ml, final 0.5 µg/ml) and incubate at 55°C for 2 h until the solution becomes clear. Add equal volume of PCI solution (Phenol:Chloroform: Isoamylalcohol=25:24:1, Merck) and mix carefully by inverting for 5 min. Centrifuge the mixture at 13,000 rpm for 5 min at 4°C. Transfer carefully the DNA-containing upper layer to new 1.5 ml E-tube. Add 2× volume of chilled 100% ethanol and 0.1× volume of 3 M sodium acetate. Precipitate the mixture at 80°C for 2 h. After precipitation, centrifuge the sample at 13,000 rpm for 30 min at 4°C. The DNA pellet was washed twice with chilled 70% (v/v) ethanol and dry the sample for 5–10 min in clean bench. Elute the DNA pellet with DNase-free water or appropriate solution for storage.

##### 1.2. Commercial kits extraction

In this method, three commercial kits were used: Bacteria DNA Preparation kit for DNA isolation (Cat. # PP-2152, Jena Bioscience), LaboPass Bacteria Mini (Cat. # CMBA0112, CosmoGenetech), and Total DNA Extraction S&V Kit (Cat. # DN40200, Bionics).

#### 2. DNA purification

DNA was purified using a column-based purification step. This method is a modification of the FastDNA^TM^ SPIN Kit for Soil (Cat. # 6560200, MP Biomedicals) protocol. The FastDNA^TM^ Spin Kit for Soil contains all the reagents required for DNA extraction in a process that efficiently removes humic acids (Has) and other PCR inhibitors. Gently mix DNA (up to 100 µl) by pipetting 10 times, add 1 ml of binding matrix suspension in sterile 1.5 ml micro tube and incubate at room temperature for 3 min. Remove 500 µl of the supernatant and resuspend the binding matrix and DNA with the remaining supernatant. Transfer the mixture into a SPIN filter and centrifuge at 13,000 rpm for 1 min at 4°C. Remove the filtrate and add 500 µl SEWS-M wash buffer. Gently resuspend by pipetting and centrifuge at 13,000 rpm for 1 min at 4°C two times. Change the collection tube to clean catch tube. Dry the SPINfilter at room temperature for 5 min in clean bench. Add 50 µl DES elution buffer (or other appropriate elution buffer) and gently resuspend the binding matrix. Incubate at room temperature for 5 min and centrifuge at 13,000 rpm for 1 min at 4°C.

## Materials

### A. Reagents

- Sterile sea water

- 50X TAE buffer (2 M Tris-acetate, 50 mM EDTA; pH 8.3)

- 2 µM deoxynucleotide triphosphate (dNTPs)

- 10X Ex-Taq buffer (200 mM Tris-HCl; pH 8.4, 500 mM KCl)

- Ex-Taq DNA polymerase

- 0.5 µg/µl ethidium bromide (EtBr)

- Agarose

- Absolute ethanol

- TNE buffer (100 mM Tris-HCl, 100 mM NaCl, 0.5 mM EDTA; pH 8.0)

- TE buffer (10 mM Tris-HCl, 1 mM EDTA; pH 8.0)

- 10% sodium lauryl sarcosine

- 10% SDS solution

- 10 mg/ml RNase A

- 20 mg/ml proteinase K

- Phenol-chloroform-isoamylalcohol (25:24:1) solution

- 3 M sodium acetate

- DNase-free water

- Purification buffers in FastDNA^TM^ SPIN Kit for Soil (Cat. # 6560220, MP Biomedicals): Binding Matrix suspension, SEWS-M wash buffer, DES elution buffer

### B. Equipments

- Micro-centrifuge

- Conical tube centrifuge

- Vortex mixer

- Heating block (or water bath)

- Thermal cycler

- Nanodrop Spectrophotometer ND-2000 (Thermo Fisher)

- Handy held homogenizer (Possible to replace with kitchen blender)

### C. Consumables

- 50 ml conical tubes

- 15 ml conical tubes

- 1.5 ml microcentrifuge tubes

- 3 μm pore size membrane filter

## Protocols

### A. Bacterial cell separation from host

• Note: All procedures were performed on ice.

1. Wash the surface of marine organisms twice with sterile seawater using a pipette. During the procedure, sample should be placed on ice.

• Note: The samples can be stored at –80°C in 100% ethanol before bacterial cell separation.

2. Place the 90 g of washed samples in a 250 ml beaker containing 150 ml of sterile seawater.

3. Slice the samples using a sterile mayo scissor or scalpel and homogenize using a hand mixer until any large particles disappear.

• Note: The mixture was vortexed vigorously enough to separate any microorganisms attached to the host for high recovery efficiency.

4. Filter the homogenized samples with 100 μm pore size sieve (Cisa, Spain).

5. Centrifuge the filtered samples at 250 g for 10 min at 4°C to remove host tissue debris and any other large contaminants.

6. Transfer the supernatant carefully into a new sterile tube of appropriate size.

7. Filter the obtained bacteria-containing supernatant two times with 3 μm pore size membrane filter (MF-Millipore Membrane Filters, Merck).

8. Centrifuge the filtered samples at 13,000 rpm for 10 min at 4°C.

• Note: Centrifuge time can be adjusted depending on the amounts of bacterial cells.

9. Wash the pellet with the chilled 100% ethanol until pigments were removed and stored at –80°C until DNA extraction.

### B. Metagenomic DNA extraction and purification

1. Centrifuge the isolated bacterial cells in 100% ethanol at 13,000 rpm for 10 min at 4°C. Remove the supernatant

2. Wash the pellet twice with the chilled TNE buffer (100 mM Tris-HCl, 100 mM NaCl, 0.5 mM EDTA; pH 8.0).

3. Resuspend the washed pellet with 500 µl of TNE buffer.

4. Add 10% sodium lauryl sarcosine (Final 1%), 10% SDS solution (Final 1%), 10 mg/ml RNase A (Final 0.1 mg/ml) into the cell suspension.

5. Mix gently by inverting 10 times.

6. Incubate at 37°C for 15 min in water-bath.

7. Add proteinase K (20 mg/ml, final 0.5 µg/ml).

8. Incubate at 55°C for 2 h until the solution is clear.

• Note: Incubation is possible for over-night for DNA extraction efficiency.

9. Add PCI (Phenol:Chloroform:Isoamylalcohol) solution as the same volume ant mix carefully by inverting for 5 min.

10. Centrifuge the mixture at 13,000 rpm for 5 min at 4°C.

11. Transfer the upper layer DNA-containing solution carefully to new 1.5 ml microcentrifuge tubes.

• Note: To ensure DNA quality, we recommend using about 400 µl.

12. Add 2× volume of chilled 100% ethanol and 0.1× volume of 3 M sodium acetate. Gently invert until the mixture is fully mixed and clear.

13. Precipitate the mixture at 80°C for 2 h.

14. After precipitation, centrifuge the sample at 13,000 rpm for 30 min at 4°C. Remove the supernatant.

15. Wash the DNA pellet with chilled 70% (v/v) ethanol twice.

16. Dry the sample for 5–10 min in clean bench.

17. Elute the DNA pellet with DNase-free water or appropriate solution for storage.

18. Gently mix by pipetting 10 times the extracted DNA (up to 100 µl) and 1 ml of binding matrix suspension in 1.5 ml Eppendorf tubes.

19. Incubate at room temperature for 3 min.

20. Remove the 500 µl of supernatant.

21. Resuspend the binding matrix and DNA with remaining supernatant.

22. Transfer the mixture into SPIN filter and centrifuge at 13,000 rpm for 1 min at 4°C.

23. Remove the filtrate and add 500 µl of SEWS-M wash buffer.

24. Gently resuspend by pipetting and centrifuge at 13,000 rpm for 1 min at 4°C. This wash step was performed two times.

25. Change the collection tube to clean catch tube.

26. Dry the tube at room temperature for 5 min in clean bench.

27. Add the appropriate elution buffer and gently resuspend the binding matrix.

28. Incubate at room temperature for 5 min and centrifuge at 13,000 rpm for 1 min at 4°C.

## Expected Results

**Fig. 3.**
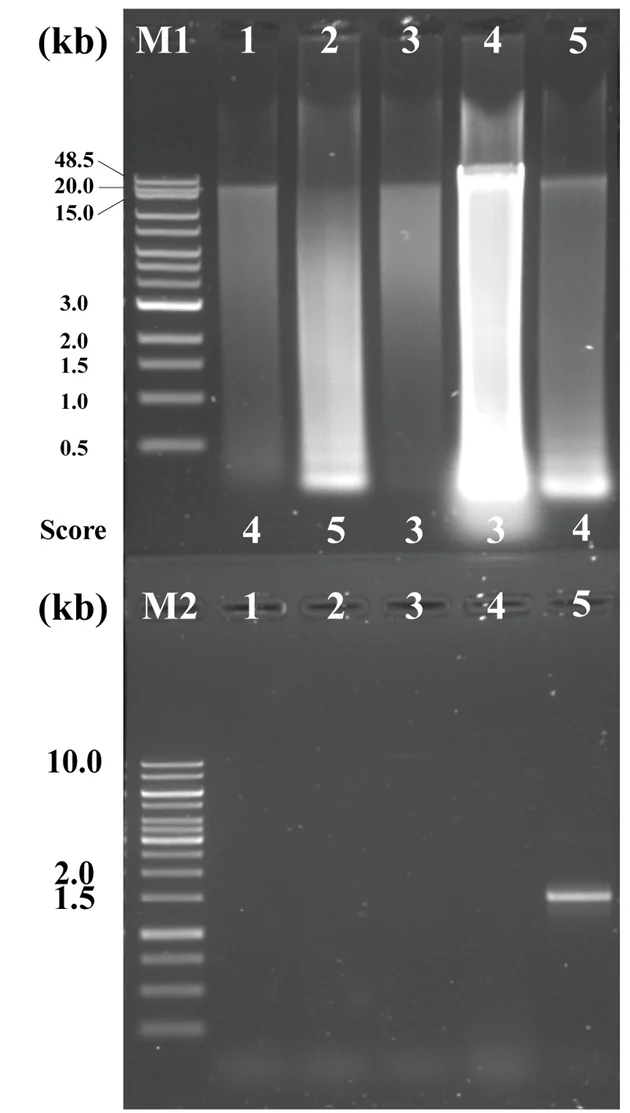
The extracted DNA from sponge-associated bacteria with the described protocols, three kits and standard manual extraction method. Upper agarose gel contained the genomic DNA, and the electrophoresis was performed at 100 V for 50 min on 0.6% agarose in 0.5X TAE buffer. The lower agarose gel contained the each 16S rDNA PCR products using the genomic DNA as a template. The gel was electrophoresed at 120 V for 30 min on 0.8% agarose in 0.5X TAE buffer. M1: Quick-Load 1 kb Extend DNA Ladder (NEB); M2: GeneRuler 1 kb DNA Ladder (Thermo Scientific^TM^); Lane 1: DNA using Jena Bioscience kit; Lane 2: DNA using Cosmo kit; Lane 3: DNA using Bionics kit; Lane 4: Standard manual extracted DNA; Lane 5: Column-based purified DNA after manual extraction.

In this study, the genomic DNA was extracted by two different methods (extraction by commercial kits (three different) vs. standard manual extraction). (A) Bacteria Preparation kit for DNA isolation (Jena Bioscience), (B) LaboPass Bacteria Mini (CosmoGenetech), and (C) Total DNA Extraction S&V Kit (Bionics) were used for bacterial DNA isolation. The DNA samples were electrophoresed on a 0.6% agarose gel in Tris-acetic acid-EDTA (TAE) buffer at 100 V for 50 min to compare the degree of damage and major band size. The agarose gel was stained with Ethidium bromide (EtBr) solution (0.5 µg/µl) for 15 min and de-stained 30 min with TDW. Nanodrop Spectrophotometer ND-2000 (Thermo Fisher) was used to assess the quality and quantity. Each DNA sample was eluted in a final volume of 50 µl, and 10 µl of the eluate was used for electrophoresis. To compare DNA extraction efficiency, yields were normalized to cell suspensions equivalent to OD_600_=1.0 in 1 ml volume. As a result, using the three kits, the efficiencies were (A) 0.733 µg, (B) 1.045 µg, and (C) 0.131 µg (Table 1). Among the kits, LaboPass Bacteria Mini kit (CosmoGenetech) was more efficient for genomic DNA isolation than other two kits. However, the DNA extracted with CosmoGenetech kit was almost sheared, whereas a little large size DNA band about 20 kb was observed in two other samples (Fig. 3). The standard manual extraction had the highest extraction efficiency that was 3.161 µg of DNA per OD_600_ = 1 × 1 ml cell and the large size of DNA band about 48.5 kb was observed, clearly. The samples had similar quality of DNA; A_260/280_ ratio was about 1.82–2.07 (Table 1). To evaluate the quality of the genomic DNA, the score given in Amos and Hoelzel (1991) was used (Fig. 2). Among the samples, manual extracted DNA sample had the best score (score 3), whereas three kits ([A] Jena Bioscience, [B] CosmoGenetech, and [C] Bionics) extracted DNA samples had 20 kb or lower and scored 4, 5, 4, respectively. The largest size DNA band was observed in manually extracted DNA and Jena kit extracted DNA. The gDNA using Cosmo kit was almost sheared and observed RNA contamination. Considering the amount and size of DNA recovered from bacterial cells, it was confirmed that standard manual extraction method was the suitable method. In addition, PCR test using each 250 ng DNA was failed in all four samples possibly due to the presence of some contaminants, such as PCR inhibitors, in the eluted DNA samples. To improve the quality, an additional column-based purification was performed on the manually extracted DNA. The purified DNA using column had a large size DNA band about 20 kb. However, the size of DNA decreased from about 48.5 kb to 20 kb. After purification, the PCR product band corresponding to 16S rRNA gene was observed using the purified gDNA as a template. It was indicated that the contaminants were removed through the column-based purification (Fig. 3).

**Fig. 4.**
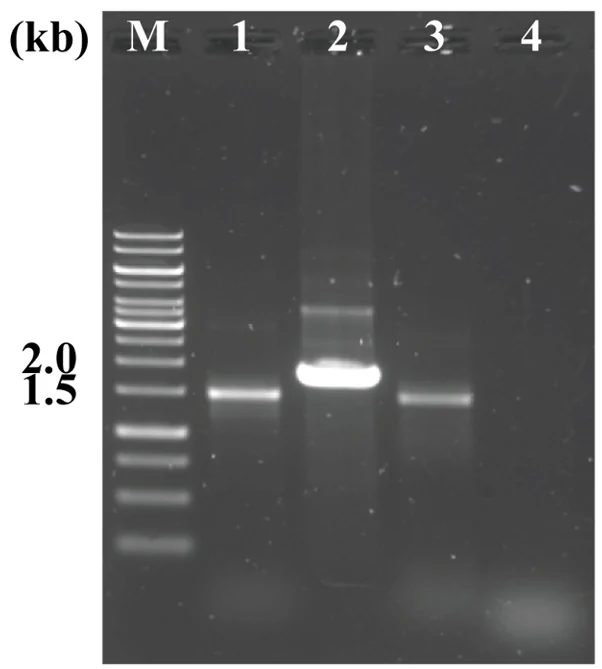
The effect of membrane filter on host tissue filtering. M: GeneRuler 1 kb DNA Ladder (Thermo Scientific^TM^, 200 ng); Lanes 1 and 2: 16S and 18S rDNA PCR products from non-filtered bacterial cells; Lanes 3 and 4: 16S and 18S rDNA PCR products from the filtered bacterial cells. The electrophoresis was performed at 120 V for 30 min on a 0.8% agarose gel in TAE buffer. All samples were prepared using purified manually extracted DNA.

To identify the effect of membrane filter on host tissue elimination, an additional filtration with 3 μm pore size membrane filter was carried out to separate the bacterial cells from the eukaryotic host tissue cell. The 16S and 18S rRNA genes targeting PCR tests were performed using the appropriate primer sets to confirm that eukaryotic host cells were removed. The metagenomic DNA was extracted by standard manual extraction procedure with and without membrane filtration process, respectively. Both genomic DNA samples were purified with column-based purification because the extracted DNA had not enough quality for PCR test. As a result, both 16S and 18S rRNA genes were amplified from gDNA extracted without a 3 μm pore size membrane filtration process, whereas only 16S rRNA gene was detected from the gDNA extracted via process include 3 μm pore size membrane filtration. This result indicated that to remove host tissue cells, filtration process with 3 μm pore size membrane filter should be included (Fig. 4).

To remove large host tissue cells, a low-speed centrifugation step (250 g for 10 min) was performed, although some microbial cell loss during this process is inevitable. While optical density (OD_600_) measurements before and after centrifugation were not conducted, visual inspection suggested that the difference was not significant. Microbes tightly associated with host cells are likely to be lost at this stage. To minimize such loss and maximize recovery of bacterial cells, homogenization (Protocol step A-3) was followed by vigorous vortexing. Repeating these step multiple times, especially for large sample volumes, can further reduce bacterial cell loss. Centrifugation and membrane filtration, which are the key steps used to remove host cells in this protocol, can affect microbial community composition and diversity indices, including species richness, abundance, and detection of rare taxa. Meanwhile, differential low-speed centrifugation tends to remove relatively heavy and high-abundance species, which may facilitate the detection of rare microorganisms (Macher et al., 2019). However, this can also cause loss of dominant taxa, introducing bias in abundance-based interpretations. Similarly, pore size of membrane affects taxonomic profiles and relative abundance estimates: smaller pores enrich free-living bacteria, while larger pores allow particle-associated or aggregated cells to pass through (Byappanahalli et al., 2021). Using a 3 μm pore size membrane filter, as in this study, is expected to remove particle-associated microbes, potentially affecting the abundance and diversity. Therefore, selecting pore size according to the target microbial groups is important, and this protocol provides flexibility in pore size control, which can help detect the microbial community that suits the purpose.

In conclusion, the application of this protocol for sample preparation enables successful metagenomic sequencing with minimal host-derived reads. The efficiency of host DNA removal can be validated by mapping sequencing reads to the host reference genome. The high-quality dataset generated through the protocol described in this study will offer valuable genetic information for future research in metagenomics, metatranscriptomics, and host transcriptome analysis.

## Figures and Tables

**Table 1.** Influence of extraction methods on the yield, concentration, and quality of DNA. The assessment was performed by measuring the concentration, A_260/280_, and A_260/230_ values using Nanodrop spectrophotometry. The yield (%) of additional column-based purification from manually extracted DNA was calculated.

Methods	Concentration (ng/µl)	A_260/280_	A_260/230_	DNA yield (µg) per OD_600_=1.0 per 1 ml bacterial cells	DNA purification yield (%)
Kit1 (Jena Bioscience)	58.6	1.82	0.22	0.733	-
Kit2 (Cosmo)	83.6	1.86	0.34	1.045	-
Kit3 (Bionics)	15.3	2.07	15.51	0.131	-
Standard manual extraction	991.5	1.93	1.54	3.161	-
Column-based purified DNA	104.5	1.85	0.18	-	79.05
